# DNA Methylation Levels of Melanoma Risk Genes Are Associated with Clinical Characteristics of Melanoma Patients

**DOI:** 10.1155/2015/376423

**Published:** 2015-04-12

**Authors:** Érica S. S. de Araújo, Dimitrius T. Pramio, André Y. Kashiwabara, Paula C. Pennacchi, Silvya S. Maria-Engler, Maria I. Achatz, Antonio H. J. F. M. Campos, João P. Duprat, Carla Rosenberg, Dirce M. Carraro, Ana C. V. Krepischi

**Affiliations:** ^1^International Research Center, A.C.Camargo Cancer Center, Rua Taguá 440, 01508010 São Paulo, SP, Brazil; ^2^Federal Technological University of Paraná, Avenida Alberto Carazzai 1640, 86300000 Cornélio Procópio, PR, Brazil; ^3^School of Pharmaceutical Sciences, University of São Paulo, Avenida Professor Lineu Prestes 580, 05508000 São Paulo, SP, Brazil; ^4^Department of Oncogenetics, A.C.Camargo Cancer Center, Rua Professor Antônio Prudente 211, 01509010 São Paulo, SP, Brazil; ^5^Department of Pathology, A.C.Camargo Cancer Center, Rua Professor Antônio Prudente 211, 01509010 São Paulo, SP, Brazil; ^6^Skin Cancer Department, A.C.Camargo Cancer Center, Rua Professor Antônio Prudente 211, 01509010 São Paulo, SP, Brazil; ^7^Department of Genetics and Evolutionary Biology, Institute of Biosciences, University of São Paulo, Rua do Matão 277, 05508090 São Paulo, SP, Brazil

## Abstract

In melanoma development, oncogenic process is mediated by genetic and epigenetic mutations, and few studies have so far explored the role of DNA methylation either as predisposition factor or biomarker. We tested patient samples for germline *CDKN2A* methylation status and found no evidence of inactivation by promoter hypermethylation. We have also investigated the association of clinical characteristics of samples with the DNA methylation pattern of twelve genes relevant for melanomagenesis. Five genes (*BAP1, MGMT, MITF, PALB2*, and *POT1*) presented statistical association between blood DNA methylation levels and either *CDKN2A*-mutation status, number of lesions, or Breslow thickness. In tumors, five genes (*KIT, MGMT, MITF, TERT*, and *TNF*) exhibited methylation levels significantly different between tumor groups including acral compared to nonacral melanomas and matched primary lesions and metastases. Our data pinpoint that the methylation level of eight melanoma-associated genes could potentially represent markers for this disease both in peripheral blood and in tumor samples.

## 1. Introduction

Cutaneous melanoma has an increasing incidence rate worldwide [1]. The occurrence of this type of cancer is associated with skin color, geographic localization, and intermittent exposure to sunlight, and the former is probably the major cause of the increase in melanoma cases [2].

The risk of melanoma development rises 30- to 70-fold in individuals with family history of the disease [3]. Two genes harboring high-risk germline mutations have been recognized:* CDKN2A* [4–6] and* CDK4* [7]. Germline mutations in additional genes were recently associated with melanoma occurrence:* MITF* [8],* BAP1* [9],* TERT* promoter [10],* POT1* [11], and* MGMT* [12]. In addition, some alterations in genes known to be related to melanin synthesis, such as* MC1R *[13, 14], have been reported as low-risk variants for melanoma development. The implication of other candidate genes in melanoma predisposition is supported by less conclusive evidence, such as* PALB2*, in which a deleterious mutation has been detected in a single individual affected by four kinds of cancer, including melanoma [15].

In addition to genetic mutations, early events of anomalous DNA methylation at specific* loci* (epimutations) can eventually result in cancer predisposition, as already reported for colorectal [16, 17], gastric [18], and breast cancers [19]. Although melanoma epimutations have not been identified so far [20, 21], abnormal methylation levels of* TNF* and* TNFRSF10C* genes have been associated with melanoma risk [22, 23]. Moreover, somatic DNA methylation changes are crucial for skin melanoma progression [24]. Recently, the occurrence of* KIT* epigenetic silencing in cutaneous melanomas has been demonstrated [25], supporting earlier studies that had shown decrease of* KIT* expression associated with melanoma progression [26].

In this work, we investigated the methylation pattern of germline* CDKN2A* and other 10 melanoma-related genes in leukocytes of melanoma patients (familial and sporadic cases), relating these findings to melanoma occurrence and clinical characteristics. The methylation profile of these genes and* KIT* was also investigated in a group of cutaneous melanomas.

## 2. Material and Methods

This was a retrospective study based on samples from melanoma patients retrieved from the A.C.Camargo Cancer Center Biobank, São Paulo, Brazil, and approved by the Institutional Ethics Committee Board (1695/12 and 1765/13).

### 2.1. Peripheral Blood Samples of Melanoma Patient and Control Groups

Melanoma patients were treated at the A.C.Camargo Cancer Center, São Paulo, Brazil. DNA samples were extracted from peripheral blood obtained from 69 unrelated cutaneous melanoma patients who had not previously received chemotherapy, most of them being stage I (58.0%). The patients were classified into three groups: hereditary melanoma carrying pathogenic* CDKN2A*-mutations (*n* = 8), familial history of melanoma without* CDKN2A*-mutations (*n* = 20), and sporadic cases (without family history of the disease; *n* = 41). The 20 hereditary melanoma patients who had previously tested negative for* CDKN2A*-mutations [27] fulfilled at least one of the following criteria: (a) familial melanoma (family history of three or more relatives of two consecutive generations with melanomas, at least one case before 50 years old; *n* = 9) or (b) multiple primary melanomas (≥3 primary lesions, *n* = 8; or ≥2 primary melanomas, one of which before 35 years old, *n* = 3).

The control group was composed of 12 individuals without cancer history, matched by age (range for each being 10 years) and gender to the subset of melanoma patients who were analyzed by DNA methylation array.

### 2.2. Cutaneous Melanoma and Melanocyte Samples

Frozen tissues from 18 unrelated primary skin melanomas (five acral and thirteen nonacral subtypes) were retrieved from the A.C.Camargo Tissue Bank. DNA extraction was processed at A.C.Camargo DNA and RNA Bank [28]. Paired metastases were available for seven tumors. The majority of tumors were classified as stage III (66.7%), but this information was missing for the remaining samples.

The primary cultures of melanocytes were provided by the School of Pharmaceutical Sciences (University of São Paulo). Primary skin cell cultures (melanocytes) were obtained from foreskin samples of three healthy donors [29] under approval of the local ethics committee (CEP HU/USP 943/09).

### 2.3. Pyrosequencing

Quantitative bisulfite pyrosequencing for* CDKN2A *promoter was performed using PyroMark Q96 CpG p16 (Qiagen), which analyzes seven CpG sites of p16 isoform (position +148 to +174 of the ENSG00000147889). A total of 68 peripheral blood samples from patients were analyzed: seven* CDKN2A*-mutation carriers, 20 hereditary melanoma patients without* CDKN2A*-mutations, and 41 sporadic melanoma patients. The methylation levels were obtained using PyroMark Q96-CpG Software (Qiagen), which provides the percentage of methylated cytosines relative to the sum of methylated and unmethylated cytosines.

### 2.4. HM450K Genome-Wide Methylation Analysis

DNA methylation array was performed in samples from 18 cutaneous melanomas, three melanocyte primary cultures, and leukocyte samples from 12 control individuals and from a subset of 39 melanoma patients (eight* CDKN2A*-mutation carriers, 19 hereditary melanoma patients without* CDKN2A*-mutations, and 12 sporadic melanoma patients).

Bisulfite modification was performed on 1 *μ*g of DNA using EZ DNA methylation kit (Zymo Research). The DNA methylation levels of CpG sites were obtained using the Infinium HumanMethylation450K BeadChips (HM450K, Illumina), according to the manufacturer's instructions. Bioconductor IMA package was applied for quality control of array methylation data [30]. We removed from further analyses probes that were lacking beta-values, containing SNPs, mapped at chromosomes X and Y, or with detection *P* values > 0.01. Probe type bias adjustment was achieved by Beta Mixture Quantile dilation (BMIQ) using ChAMP package [31]. 

The level of methylation of each CpG probe was calculated as a beta-value ranging from 0 to 1 (0 for totally unmethylated CpG sites and 1 for fully methylated sites). Beta-values were retrieved for all CpG probes mapped within DNA sequences associated with the* BAP1, CDK4*,* CDKN2A*,* KIT*,* MC1R, MGMT*,* MITF*,* PALB2*,* POT1*,* TERT*,* TNF,* and* TNFRSF10C *genes (see Supplementary Tables  1 and 2 in Supplementary Material available online at http://dx.doi.org/10.1155/2015/376423). CpG sites were grouped into two categories, according to the annotation of the transcripts provided by Illumina, which is based on the UCSC database: “promoter” region (CpG probes mapped at TSS1500, TSS200, 5′UTR, or 1st* exon*) and “gene body” (remaining exons and intron probes). Methylation levels for promoters and bodies were obtained using the beta-value means of the corresponding CpG probes. 

Microarray methylation data from peripheral blood were deposited into NCBI's Gene Expression Omnibus under accession number GSE54939 (http://www.ncbi.nlm.nih.gov/geo/query/acc.cgi?acc=GSE54939).

### 2.5. Statistical Analysis of HM450K Data

DNA methylation differences between groups were tested for significance using either Mann-Whitney test or Kruskal-Wallis with Dunn's Multiple Comparison Post-test. The analysis comparing primary cutaneous melanoma and paired metastasis was performed using paired *t*-test.

## 3. Results

The quantitative bisulfite pyrosequencing of* CDKN2A* promoter was investigated to determine if any of the melanoma patients exhibited inactivation of this gene by hypermethylation. Data showed no evidence of* CDKN2A* hypermethylation in leukocytes of these patients. Among* CDKN2A*-mutation carriers, the methylation mean was 0.63% (ranging from 0 up to 1.5%), hereditary melanoma patients without* CDKN2A-*mutations showed a mean of 0.52% (0–3.0%), and sporadic melanoma patients presented a mean of 0.35% (0–1.5%). Statistical association between the clinical characteristics of melanoma patients and the methylation levels of p16 encode region was not detected (Breslow thickness *P* = 0.78; number of melanomas *P* = 0.84).

Next, we compared the levels of DNA methylation for CpG sites mapped in eleven melanoma-associated genes (*BAP1, CDK4*,* CDKN2A*,* MC1R, MGMT*,* MITF*,* PALB2*,* POT1*,* TERT*,* TNF,* and* TNFRSF10C*) between melanoma patients and controls. Only the gene body of* MGMT* showed a low level of methylation increase in* CDKN2A*-mutated patients compared to sporadic melanoma patients (2% increase in the methylation level, *P* = 0.02, Supplementary Table 3). Additionally, DNA methylation levels at the promoter regions of* BAP1*,* MITF,* and* PALB2* genes were statistically associated with the number of melanomas presented by the individual and a hypermethylation of* POT1 *promoter was associated with Breslow thickness of the tumors (Table 1).

We also investigated the DNA methylation levels of twelve melanoma-associated genes (*BAP1, CDK4*,* CDKN2A*,* KIT*,* MC1R, MGMT*,* MITF*,* PALB2*,* POT1*,* TERT*,* TNF*, and* TNFRSF10C*) by comparing melanoma samples with cultured melanocytes. The methylation levels at the gene bodies of* MGMT*,* TERT,* and* TNF* were significantly lower in tumors, while both body and promoter of* KIT* were hypermethylated (Table 2). Acral melanomas exhibited differential methylation at the gene bodies of* MGMT* (4% hypomethylation in acral melanoma group, *P* = 7*e* − 3, Supplementary Table 4) and* TERT* (9% hypermethylation in acral melanoma group, *P* < 1*e* − 3, Supplementary Table 4) compared to nonacral tumors. We did not find significant differences comparing tumors from patients who did develop or not metastases (Supplementary Table 5); however, in the analysis of the seven paired primary-metastasis melanoma samples, the gene body of* MITF* was found to be hypomethylated in metastasis (8% of decreased methylation in relation to primary tumor, *P* = 0.02, Supplementary Table 6).

## 4. Discussion

Aberrant DNA methylation is usually an early and stable event in tumorigenesis and could be used for detecting and monitoring diseases [32]. Previous studies in melanoma and surrogate samples have revealed epigenetic markers based on methylation patterns of* LINE-1* [33, 34]*, RASSF1A* [35],* TSLC1 *[36],and* MGMT *[37]. In this study, we have investigated the methylation levels of twelve melanoma genes to evaluate their potential role as predisposition factors and/or biomarkers. In fact, control of gene expression by DNA methylation has already been described for half of these investigated genes in different types of cancer [38–43]. Considering that the functional impact of DNA methylation in gene expression is better understood for CpGs mapped at gene promoters, we analyzed separately the probes mapped at promoters and gene bodies. Most of the genes here analyzed exhibited CpG islands in their promoter sequences, except for* MITF* and* TNF*. 

Independent cohorts of patient peripheral blood and melanoma samples were analyzed. Overall, the detected DNA methylation differences in blood were very small (<5%), while tumors exhibited higher (>10%) differences. Indeed, previous studies have shown that epigenetic biomarkers in peripheral blood exhibit very small differences [44–47]. Significant DNA methylation changes were identified in peripheral blood mainly at gene promoters, while in tumors they appeared to cluster at gene bodies. Also, melanomas were mainly hypomethylated when compared to the control melanocytes; however, we cannot exclude the possibility that the DNA methylation pattern of the melanocytes could be altered because of* in vitro* conditions [48].

*MGMT* encodes a repair protein which removes alkyl groups from the O_6_-position of guanine residues [49] and its promoter methylation has been described as a biomarker in several studies, especially for glioblastoma [50] and colorectal cancer [51]. In melanoma, epigenetic silencing of this gene has been demonstrated in tumors and serum of patients [52, 53], suggesting that this event would be important for tumor development. In our study, we detected methylation differences at* MGMT* body both in peripheral blood and in tumor samples:* CDKN2A*-mutated patients showed a low-level hypermethylation in leukocytes compared to other melanoma patients, while tumors showed hypomethylation compared to melanocytes. Even though gene body methylation is a yet poorly explored mechanism, it has been reported in association with increased gene expression [54]. However, the relationship between intragenic methylation and gene expression appears not to be linear, since low body methylation levels have been associated with both low and high expression, and high body methylation levels have been already reported linked to intermediate expression [55]. Additional studies in melanomas are necessary to clarify the role of the observed methylation changes in the expression of the* MGMT* gene.

*MITF* is a transcription factor that controls genes of cell cycle and melanogenesis [56, 57]. Hypermethylation of the* MITF* promoter was detected in peripheral blood of those melanoma patients who developed more than one lesion (multiple primary melanomas), and* MITF* gene body was found to be somehow hypermethylated in primary tumors compared to metastasis. Interestingly,* MITF* expression was reported as variable among melanoma specimens and even intratumor [58], with high expression levels being associated with either differentiation or proliferation while low expression is related to an invasive potential [59]. This dual role of* MITF* according to its expression level could be involved in the different patterns of methylation observed in peripheral blood and tumors, suggesting that (a) high methylation levels at* MITF* promoter in leukocytes could be linked to a more aggressive disease and (b) higher methylation level at* MITF* gene body of primary tumors compared to metastasis would have a controlling role in cell cycle. Lauss and colleagues [60] have recently shown that* MITF* expression in melanomas is controlled by DNA methylation, but unfortunately RNA was unavailable to investigate whether the low DNA methylation changes here detected would impact* MITF* expression.

We also detected DNA methylation differences between acral and nonacral melanoma subtypes at* MGMT* and* TERT* bodies. Acral melanoma is an uncommon subtype not associated with sunburn and occurs more frequently in older and non-Caucasian individuals [61]. Additionally, acral driver mutations are distinct compared to cutaneous melanomas. For instance,* KIT* mutations are more common in acral than in the other melanomas [62, 63]. We did not observe significant difference in* KIT* methylation levels between acral and nonacral subtypes, but melanomas as a group were hypermethylated compared to melanocytes. The detection of* KIT* hypermethylation in the advanced tumors of this study (mostly stage III melanomas) was not unexpected and had already been described [25, 64].

The* CDKN2A* methylation level was analyzed by the use of microarray (HM450K) and pyrosequencing. This gene encodes p16 and p14 proteins, the p16 isoform harboring the majority of the germline mutations that confer a high melanoma risk [65]. Since the array platform contains* CDKN2A* CpGs not specifically associated with p16, we investigated additional* CDKN2A* promoter CpGs by pyrosequencing. The melanoma patients exhibited low methylation levels for* CDKN2A*, similar to the pattern already reported for healthy individuals [23]. Our data in this Brazilian cohort of patients are in accordance with previous studies that described absence of* CDKN2A *epimutation in hereditary melanoma patients [20, 21].

## 5. Conclusion

Here we investigated the potential role of twelve melanoma-associated genes as predisposition factors and epigenetic biomarkers for melanoma, using both peripheral blood and tumor samples. We did not find germline* CDKN2A* hypermethylation in our cohort of Brazilian melanoma patients. However, we detected low-level albeit significant DNA methylation differences in a gene subset both in leukocytes and in tumors (*BAP1*,* KIT*,* MGMT*,* MITF*,* PALB2*,* POT1*,* TERT,* and* TNF*). Additional studies in a large melanoma cohort are necessary to validate these candidate genes as epigenetic biomarkers in melanoma.

## Supplementary Material

The Supplementary Material files contain the list of the HM450K probes that were analyzed in peripheral blood samples (Supplementary Table 1) and in melanoma samples (Supplementary Table 2), the DNA methylation levels of melanoma-associated genes in peripheral blood samples (Supplementary Table 3) and in melanoma samples (Acral versus Non-acral melanomas, Supplementary Table 4; primary melanomas from patients who did develop or not metastasis, Supplementary Table 5; paired primary tumors and metastasis, Supplementary Table 6).

## Figures and Tables

**Table 1:** Methylation levels of melanoma susceptibility genes in peripheral blood of melanoma patients and association with clinical characteristics.

Clinical characteristics of patients	*N*	*BAP1 *		*CDK4 *		*CDKN2A *	*MC1R *	*MGMT *	
		Promoter	Body	Promoter	Body	Promoter	Promoter	Promoter	Body
		mean (SD)	mean (SD)	mean (SD)	mean (SD)	mean (SD)	mean (SD)	mean (SD)	mean (SD)
*Melanomas number *									
1	18	0.04 (7*e* − 3)	0.59 (0.01)	0.25 (7*e* − 3)	0.32 (0.06)	7.4*e* − 2 (0.01)	0.36 (0.01)	0.45 (8*e* − 3)	0.87 (0.02)
>1	21	0.03 (6*e* − 3)	0.59 (0.01)	0.25 (8.4*e* − 3)	0.33 (0.04)	7.6*e* − 2 (0.01)	0.36 (0.01)	0.45 (9*e* − 3)	0.88 (0.02)
*P value *		2**e** − 4	0.60	0.16	0.97	0.62	0.42	0.85	0.11
*Breslow thickness* ^*^									
<1	24	0.04 (9.5*e* − 3)	0.59 (0.02)	0.25 (8*e* − 3)	0.33 (0.04)	0.08 (0.01)	0.36 (0.01)	0.45 (8*e* − 3)	0.87 (0.02)
≥1	13	4.5*e* − 3 (6.7*e* − 3)	0.59 (0.01)	0.25 (7*e* − 3)	0.33 (0.03)	0.07 (0.01)	0.36 (0.01)	0.45 (9*e* − 3)	0.87 (0.01)
*P value *		0.84	0.36	0.69	0.69	0.36	0.45	0.19	0.45

Clinical characteristics of patients	*N*	*MITF * (NM_000248 and NM_198158)		*MITF* (NM_006722)		*MITF* (NM_198159)		*MITF* (NM_198177)	
		Promoter	Body	Promoter	Body	Promoter	Body	Promoter	Body
		mean (SD)	mean (SD)	mean (SD)	mean (SD)	mean (SD)	mean (SD)	mean (SD)	mean (SD)

*Number of melanomas *									
1	18	0.93 (0.03)	0.94 (0.02)	0.78 (0.03)	0.87 (0.02)	0.08 (0.03)	0.73 (0.01)	0.79 (0.03)	0.91 (0.02)
>1	21	0.95 (0.02)	0.95 (0.02)	0.80 (0.03)	0.88 (0.01)	0.08 (0.03)	0.73 (0.01)	0.80 (0.02)	0.92 (0.01)
*P value *		**0.01**	0.83	**0.02**	0.10	1.00	5.4*e* − 2	**0.02**	0.20
*Breslow thickness* ^*^									
<1	24	0.95 (0.03)	0.95 (0.02)	0.80 (0.03)	0.88 (0.02)	0.08 (0.02)	0.73 (0.01)	0.80 (0.03)	0.91 (0.02)
≥1	13	0.95 (0.02)	0.94 (0.02)	0.79 (0.03)	0.87 (0.01)	0.08 (0.03)	0.73 (0.01)	0.79 (0.02)	0.91 (0.01)
*P value *		0.99	0.19	0.42	0.44	0.94	0.44	0.54	0.51

Clinical characteristics of patients	*N*	*MITF* (NM_198178)		*PALB2 *		*POT1* (NM_001042594)		*POT1* (NM_015450)	
		Promoter	Body	Promoter	Body	Promoter	Body	Promoter	Body
		mean (SD)	mean (SD)	mean (SD)	mean (SD)	mean (SD)	mean (SD)	mean (SD)	mean (SD)

*Number of melanomas *									
1	18	0.94 (0.02)	0.92 (0.02)	0.24 (9*e* − 3)	0.06 (0.01)	0.95 (0.01)	0.23 (0.01)	0.17 (0.01)	0.95 (0.02)
>1	21	0.93 (0.02)	0.93 (0.01)	0.23 (9*e* − 3)	0.07 (0.01)	0.96 (0.02)	0.23 (0.01)	0.16 (0.01)	0.96 (0.01)
*P value *		0.68	0.09	**0.01**	0.47	0.18	0.68	0.54	0.10
*Breslow thickness* ^*^									
<1	24	0.94 (0.02)	0.92 (0.02)	0.24 (9*e* − 3)	0.07 (0.01)	0.95 (0.02)	0.23 (0.01)	0.16 (0.01)	0.96 (0.01)
≥1	13	0.92 (0.03)	0.92 (0.01)	0.23 (9*e* − 3)	0.06 (0.01)	0.96 (0.01)	0.24 (0.01)	0.17 (0.01)	0.96 (0.01)
*P value *		0.26	0.76	0.81	0.26	0.58	0.06	**0.04**	0.76

Clinical characteristics of patients	*N*	POT1 (NR_003102. NR_003103 and NR_003104)		*TERT *		*TNFRSF10C *		*TNF *	
		Promoter	Body	Promoter	Body	Promoter	Body	Promoter	Body
		mean (SD)	mean (SD)	mean (SD)	mean (SD)	mean (SD)	mean (SD)	mean (SD)	mean (SD)

*Melanomas number *									
1	18	0.17 (0.01)	0.50 (0.01)	0.58 (0.039)	0.86 (0.02)	0.38 (0.01)	0.12 (0.02)	0.21 (0.03)	0.67 (0.05)
>1	21	0.17 (0.01)	0.50 (0.02)	0.58 (0.043)	0.86 (0.01)	0.38 (0.01)	0.11 (0.02)	0.22 (0.03)	0.69 (0.07)
*P value *		0.75	0.72	0.75	0.13	0.15	0.94	0.32	0.33
*Breslow thickness* ^*^									
<1	24	0.16 (0.01)	0.50 (0.02)	0.58 (0.046)	0.86 (0.02)	0.38 (0.01)	0.11 (0.02)	0.22 (0.03)	0.69 (0.07)
≥1	13	0.17 (0.01)	0.51 (0.01)	0.58 (0.028)	0.86 (0.01)	0.38 (0.01)	0.12 (0.02)	0.21 (0.03)	0.66 (0.05)
*P value *		0.07	0.12	0.54	0.35	0.79	0.54	0.74	0.35

*N*: number of samples; promoter: probes mapped at TSS1500, TSS200, 5′UTR, or 1st exon; mean of DNA methylation levels; SD: standard deviation; ^*^Breslow thickness of high melanoma stage; values highlighted in bold are statistically significant (*P* < 0.05. Mann-Whitney test).

**Table 2:** Methylation levels of melanoma susceptibility genes in primary melanomas and melanocyte primary cultures from healthy donors.

Gene name	Chromosome	UCSC accession (transcripts)	Genic region	CpG loci number	Melanomas (SD)	Melanocytes (SD)	*P value *
*BAP1 *	3	NM_004656	Body	5	0.52 (0.44)	0.44 (0.46)	1.00
		NM_004656	Promoter	10	0.04 (0.01)	0.05 (0.02)	0.16

*CDK4 *	12	NM_000075	Body	2	0.18 (0.09)	0.06 (0.02)	—
		NM_000075	Promoter	13	0.22 (0.30)	0.22 (0.30)	0.54

*CDKN2A *	9	NM_058195	Promoter	4	0.12 (0.03)	0.08 (0.05)	0.20

*KIT *	4	NM_000222, NM_001093772	Body	9	0.50 (0.30)	0.17 (0.28)	**0.01**
			Promoter	5	0.32 (0.24)	0.08 (0.08)	**0.03**

*MC1R *	16	NM_002386	Promoter	9	0.27 (0.32)	0.07 (0.05)	0.67

*MGMT *	10	NM_002412	Promoter	14	0.42 (0.35)	0.44 (0.42)	0.99
		NM_002412	Body	99	0.74 (0.15)	0.83 (0.20)	**<1** **e** − 4

*MITF *	3	NM_000248	Promoter	3	0.53 (0.09)	0.06 (0.05)	0.1
		NM_000248	Body	5	0.79 (0.20)	0.57 (0.45)	0.42
		NM_006722	Promoter	4	0.79 (0.10)	0.80 (0.16)	0.89
		NM_006722	Body	20	0.78 (0.17)	0.64 (0.38)	0.78
		NM_198158	Promoter	3	0.53 (0.09)	0.06 (0.05)	0.10
		NM_198158	Body	5	0.79 (0.20)	0.57 (0.45)	0.42
		NM_198159	Promoter	5	0.04 (8.4*e* − 3)	0.04 (1.1*e* − 3)	0.55
		NM_198159	Body	29	0.67 (0.29)	0.56 (0.39)	0.77
		NM_198177	Promoter	4	0.77 (0.16)	0.59 (0.29)	0.20
		NM_198177	Body	14	0.77 (0.19)	0.61 (0.42)	0.84
		NM_198178	Promoter	2	0.94 (0.04)	0.97 (0.01)	—
		NM_198178	Body	10	0.73 (0.20)	0.48 (0.43)	0.28

*PALB2 *	16	NM_024675	Body	5	0.22 (0.40)	0.22 (0.39)	0.84
		NM_024675	Promoter	12	0.07 (0.08)	0.08 (0.09)	0.37

*POT1 *	7	NM_001042594	Body	1	0.95 (0.04)	0.94 (0.01)	—
		NM_001042594	Promoter	10	0.12 (0.23)	0.14 (0.26)	0.31
		NM_015450	Body	1	0.95 (0.04)	0.94 (0.01)	—
		NM_015450	Promoter	10	0.12 (0.23)	0.14 (0.26)	0.31
		NR_003102, NR_003103, NR_003104	Body	3	0.33 (0.53)	0.33 (0.52)	0.70
		NR_003102, NR_003103, NR_003104	Promoter	7	0.16 (0.27)	0.19 (0.30)	0.71

*TERT *	5	NM_198255, NM_198253	Body	61	0.67 (0.13)	0.71 (0.19)	**0.03**
		NM_198253, NM_198255	Promoter	6	0.52 (0.24)	0.38 (0.24)	0.49

*TNFRSF10C *	8	NM_003841	Promoter	13	0.54 (0.17)	0.47 (0.32)	0.64
		NM_003841	Body	3	0.47 (0.13)	0.34 (0.38)	0.70

*TNF *	6	NM_000594	Body	5	0.76 (0.06)	0.89 (0.07)	**0.01**
		NM_000594	Promoter	13	0.54 (0.17)	0.47 (0.32)	0.64

SD: standard deviation; promoter: probes mapped at TSS1500, TSS200, 5′UTR, or 1st exon; values highlighted in bold are statistically significant (*P* < 0.05, Mann-Whitney test).
